# Promising Practices of Out‐of‐School Time Programs for Low‐Income Adolescents: A Systematic Review

**DOI:** 10.1002/jad.12506

**Published:** 2025-04-24

**Authors:** Rebecca S. Levine, Samantha Viano

**Affiliations:** ^1^ Department of Education Studies University of California San Diego CA USA; ^2^ College of Education and Human Development George Mason University Fairfax VA USA

## Abstract

**Introduction:**

Out‐of‐school time (OST) programs can have a positive impact on youth outcomes, including academic achievement and social‐emotional development. However, there are vast inequities in program accessibility and quality, with low‐income adolescents being particularly underserved. This study synthesizes research on OST programs serving low‐income adolescents in the United States to identify promising practices in staffing, participant recruitment, and participant engagement, emphasizing approaches that foster positive youth development (PYD).

**Methods:**

A systematic literature review was conducted using PRISMA guidelines, incorporating searches of three academic databases and supplementary sources. After applying inclusion and exclusion criteria, 118 studies representing 100 discrete OST programs serving adolescents (aged 11–18) were included. Data were analyzed to identify promising OST program practices.

**Results:**

Key findings reveal strategies for hiring, training, and retaining staff, including the importance of strong leadership, professional development, and community connections. Effective participant recruitment strategies leverage existing relationships with trusted adults and peers, communicate program benefits such as skill‐building and social connection, address barriers like transportation and cost, and deliver programming in accessible, familiar community spaces. The review also yielded seven engagement strategies—agency, relevance, competence, belonging, exposure, and safety/wellness—which align closely with PYD and are promoted through developmentally appropriate and culturally responsive activities, pedagogies and policies.

**Conclusion:**

By illuminating the “how” of OST program success, this review contributes to the growing body of knowledge of practices that can promote equity and positive development for underserved youth. Future research is needed to explore and refine strategies for reaching and supporting diverse adolescent populations.

Recent events, such as the COVID‐19 pandemic, have exacerbated inequities in academic and social‐emotional development, particularly for low‐income adolescents. School closures were most harmful to the poorest students, who are disproportionately Black, Latinx, and/or Indigenous, as wealthy families had more resources to dedicate to virtual school (Agostinelli et al. 2020). Additionally, mental health challenges for low‐income youth increased due to isolation (Deolmi and Pisani 2020) and financial stress (Kim et al. 2022), with developmental psychologists worried about long‐term academic and psychosocial effects. In response, some states are increasing spending for out‐of‐school time (OST) programs (California Department of Education 2022) with the hope that these programs can help build youth's developmental assets through opportunities for positive youth development (PYD) (Lerner et al. 2021).

OST programs are supervised, structured activities that students attend outside of school hours, including summer, before school, and after school. OST programs serving adolescents have the potential to engage youth at a pivotal time when they are developing identity and autonomy in regard to college and career, relationships, and health behaviors (Afterschool Alliance 2009). Indeed, the PYD literature, including Benson's developmental assets framework, explain how OST programs can serve as a type of external asset through which youth can build internal assets like positive values and social competencies (Benson 2003; Benson et al. 2011). These developmental assets contribute to youth thriving as indicated by academic success and stronger physical health in the short‐term, and higher education attainment, civic engagement, and occupational success in the long term (Gardner et al. 2008; Guzmán‐Rocha et al. 2017; Mueller et al. 2011; Scales et al. 2000; Taylor et al. 2005). Additionally, OST programs often center the local context as a key site in which youth can thrive (Lerner et al. 2021); in this way, OST programs are well‐positioned to offer opportunities in which youth are supported as well as provide support to their communities.

Access to and availability of quality OST programs, however, is inequitable, with opportunities particularly stratified by income and wealth (Deschenes et al. 2010a; Snellman et al. 2015a; Snellman et al. 2015b; McCombs et al. 2017). Over the past few decades, high‐income families have maintained high participation in OST programs, while program access has plummeted for low‐income families (Snellman et al. 2015a). This is not due to lack of demand; more parents than ever wish to enroll their children in OST programs, with unmet demand highest among low‐income compared to high‐income families (Afterschool Alliance 2020). Affluent families are able to subsidize their children's participation in OST programming either directly through tuition or by using their social capital to ensure local schools and communities provide these programs, and they are also more likely than low‐income families to have access to safe and reliable transportation to and from OST programs (Afterschool Alliance 2020).

Adolescents from low‐income families have systematically fewer OST opportunities compared to not only their peers from high‐income families, but also compared to younger children more generally. In 2011, only 19% of 21st Century Community Learning Centers served high school (HS) students; for comparison, approximately two‐thirds of centers served elementary students (Afterschool Alliance 2011). We also know that participation in OST programs tends to drop off between the elementary years and middle school (MS) years (Anderson‐Butcher 2005). The challenges with OST program access for youth of marginalized backgrounds are intertwined with structural inequality (e.g., cost of programming, lack of transportation) as well as deficit perspectives (e.g., assumptions about aptitude and willingness to participate). An equitable society would provide access to key developmental programs in ways that meet the needs of all youth, not only for younger children and those with financial means.

Even though the literature on OST is promising (e.g., Lauer et al. 2006; Durlak et al. 2010; Knopf et al. 2015), positive outcomes are likely only realized when students regularly attend a high‐quality program that is designed to meet their needs (McCombs et al. 2017). To make progress towards this justice‐oriented vision, the purpose of this study is to consolidate the research on OST programs that serve low‐income MS/HS students, students who are traditionally underserved by OST programs, to identify promising practices.

## 1 Relevant Literature on Out‐of‐School Time Programs

A wealth of systematic reviews and meta‐analyses examine whether OST programs lead to positive effects across a variety of outcomes. Prior reviews have often distinguished between OST programs offered after school versus over the summer. Several meta‐analyses and reviews included controlled studies assessing whether afterschool programs lead to positive impacts on students’ academic achievement and noncognitive outcomes (e.g., delinquency, self‐perceptions, and prosocial behaviors), with promising results (Durlak et al. 2010; Heath et al. 2022). Similar reviews on the effect of summer programming on mathematics and reading achievement found these programs often had significant, positive benefits that were often more pronounced in low‐income areas (Kim and Quinn 2013; Lynch et al. 2022; McCombs et al. 2019). Our study is more similar to prior reviews that conceived of OST programs as including summer and afterschool programming. Prior reviews on OST programs, broadly conceived, find positive effects of OST participation on academic achievement and STEM interest (Knopf et al. 2015; Lauer et al. 2006; Young et al. 2017).

However, the findings from other reviews on OST programs have, at times, not confirmed that students benefit from these programs. Several meta‐analyses of experimental and quasi‐experimental studies of afterschool programs have found null effects on school attendance, delinquency, and antisocial behavior (Kremer et al. 2015; Taheri and Welsh 2016; Heath et al. 2022). These reviews support the importance of attending to program quality and implementation (Kremer et al. 2015; Heath et al. 2022). Research on how to engage youth in OST programs is especially critical because when youth have higher OST engagement over longer periods of time, programs can more effectively encourage PYD (Agans et al. 2014; Bohnert et al. 2010; Gardner et al. 2008; Guzmán‐Rocha et al. 2017; Theokas et al. 2005). For instance, Agans et al. (2014) found that higher participation in OST for MS‐aged youth was associated with lower risk of substance abuse, depression, and risk behaviors. Considering the potential for positive benefits from OST programs for low‐income adolescents, it is important to establish how these programs can best reach and serve this population.

## 2 Theoretical Perspective: Positive Youth Development in Out‐of‐School Time Programs

Adolescence is a time of rapid changes in social, emotional, and physical development. As youth approach adulthood, developmental tasks include an increase in independence and decision‐making, exploration of identity, bonding with peers, and preparation for college and career (Steinberg and Morris 2001). As a constructive use of time outside of the typical school day, OST programs can foster youth development by providing opportunities for meaningful relationships with adults and peers, opportunities for autonomy and choice, and support with academic and professional preparation.

The popularity of OST programs for adolescents far pre‐dates the development of PYD, with this approach reframing the development of young people as resources to be developed as opposed to problems that need to be fixed (Damon 2004). The re‐framing of OST programs to integrate PYD involves specific shifts in mindsets from traditional, outdated views on adolescent development. For instance, previous work referred to adolescence as “fragile” and “vulnerable” to negative influence with youth capable of developing “resilience” to a hostile world (Benard 1991). In PYD, youth have internal assets, like personal characteristics and social skills, and external assets, including family and community support. OST programs can help youth to develop and strengthen these assets, not subvert or undermine them (Benson 2003; Benson et al. 2011). Additionally, adolescents were previously considered almost as islands unto themselves with an emphasis on self‐sufficiency (Damon 2004). In PYD, youth are situated within their communities including families, religious institutions, neighborhoods, and schools, with all of these relationships being part of their positive development (Lerner and Benson 2003). OST programs serve as one aspect of youth's external assets and can encourage youth to build both internal and external assets through programming and activities (Taylor et al. 2005), developing and aligning youth's strengths within their contexts (Agans et al. 2014; Gardner et al. 2008; Guzmán‐Rocha et al. 2017).

Literature on OST programs often integrates PYD to guide understanding of how OST can have a positive impact on youth development. A study by Deschenes et al. (2010b) found that OST programs must be geared toward supporting youth through developmental tasks by recognizing the assets of each young person, including their social networks and personality traits like curiosity. For youth in early adolescence, successful programs ensure time for socializing, providing opportunities to explore and try new things, and creating a firm yet adaptable structure and routine (Deschenes et al. 2010b). Relatedly, Morehouse (2009) developed a framework for MS students through OST programs which she called the Five Rs: Relationships between staff and youth and among youth; Relevance to youth's lives and interests; Reinforcement, validation and guidance as youth explore themselves and the world; Real‐life, hands‐on projects with real‐word implications; and Rigorous activities that provide challenge and opportunity for mastery. For older adolescents, more goal‐oriented, targeted content; responsibility and leadership opportunities; and attention toward post‐graduation are helpful strategies to promote their development (Deschenes et al. 2010b). This review attends exclusively to OST programs for adolescents to identify approaches that are designed with this developmental stage in mind.

## 3 Current Study

As previous reviews have focused on OST program effectiveness, we argue our study poses “critical questions” to extend the field of OST research in novel ways (Alexander 2020). Specifically, we identify promising strategies for how to reach and serve low‐income MS/HS youth, as the success of OST programs is predicated on their ability to attract, retain, and effectively engage with youth whose developmental needs are changing and complex. As such, the purpose of this study is to move beyond asking *whether* OST programs work to asking *how* OST programs work. Prior reviews have shown that OST programs can and often do work but have done little to lay out how they can work best for marginalized youth most in need of access to high‐quality OST programming.

We integrate a PYD perspective to situate OST programs as critical sites of youth asset building. PYD provides the theoretical basis for how OST can develop youth assets through supportive relationships that scaffold constructive use of time, and this review identifies how OST programs facilitate these opportunities. We are interested in a diverse array of OST programs, as PYD literature identifies as many as 40 different types of developmental assets that OST programs can foster (Benson et al. 2011). Additionally, PYD is a relevant perspective because it was largely developed in the United States, the country where this review is focused; we recognize that PYD may not be appropriate for other national or cultural contexts. To fill this purpose, we address the following research questions through a systematic review of the research literature:

1. What are promising practices for hiring, training, and retaining staff for OST programs that serve low‐income youth?
2. What are promising practices for recruiting low‐income youth to OST programs?
3. What are promising practices to effectively engage low‐income youth in OST programs?

## 4 Methods

This systematic review followed best practices as set forth by Preferred Reporting Items for Systematic Reviews and Meta‐Analyses (PRISMA; Alexander 2020; Page et al. 2021). First, on June 16th, 2022, we searched ERIC, PsycINFO, and Web of Science using the following search terms in a title and abstract search: (“out of school time” OR “after school” OR “summer”) AND (“marginalized” OR “vulnerable” OR “disadvantaged” OR “minorit*“ OR “disenfranchised” OR “poverty” OR “low income” OR “low SES” OR “low socioeconomic status” OR “underrepresented” OR “underserved” OR “under‐resourced” OR “at risk” OR “homeless” OR “housing insecure” OR “refugee” OR “foster” OR “LGBT*“ OR “justice‐involved”) AND (“middle school*“ OR “high school*“ OR “secondary” OR “adolescen*“ OR “teen*“). The first parenthetical in the search term limited the search to OST programs, the second parenthetical included terminology associated with marginalized youth, and the third parenthetical restricted the studies to adolescence. We supplemented the findings from these three databases through a hand search of all publications posted on the website for the National Institute of Out‐of‐School Time (NIOST), hosted by Wellesley College, including all issues of the Afterschool Matters Journal through Spring 2022. We limited our searches to studies published in English after December 31, 2011. Limiting the search to post‐recession increased the relevance of the studies, since the Great Recession^1^ had far‐reaching impacts on OST programming due to budget cuts and high demand (Afterschool Alliance 2012). The search yielded 1276 results (1108 articles from databases + 158 articles from NIOST).

We then used several pre‐specified inclusion and exclusion criteria. First, studies had to be written in English, and the OST program needed to be delivered in the United States. Second, we only included studies with original, empirical analysis, excluding reviews and studies that described programs without any data analysis. Third, we only included studies that were either from a peer‐reviewed journal or published as a working paper or report from a reputable organization, to limit to studies with an acceptable level of rigor.

Fourth, the studies needed to report on dimensions of OST programs that serve low‐income adolescents. The OST program could also enroll other youth who were not low‐income or were younger or older, as long as the program had targeted efforts for low‐income adolescents. Our target age range was 11–18 years old, representing most middle and high school students. We used several decision rules to more clearly define what “counts” as serving low‐income participants within the context of this systematic review. We included studies if they either: a) labeled the general population or surrounding schools or neighborhoods as low‐income (e.g., living in poverty, majority of students receiving free or reduced‐price lunch); b) used a term that is associated with low‐income status to describe the community or program participants (e.g., disadvantaged, underserved, at‐risk); c) reported that at least 40% of youth participating in the program were low‐income; and/or d) targeted youth who belong to a group highly associated with socioeconomic hardship (e.g., youth experiencing homelessness).

Fifth, we focused on OST programs that were at least 4 weeks in duration (duration could be any length longer than 4 weeks including year‐round). Lastly, if a program combined both in‐ and out‐of‐school components, we needed to be able to distinguish results related to OST in particular to include the study. For example, we excluded a working paper by Heller et al. (2013) because the program evaluated was comprised of sessions both during and after school. While interventions or programs that span in‐ and out‐of‐school time can be effective, they would be best suited for another review; the goal of this review is to center OST specifically.

**Figure 1:**
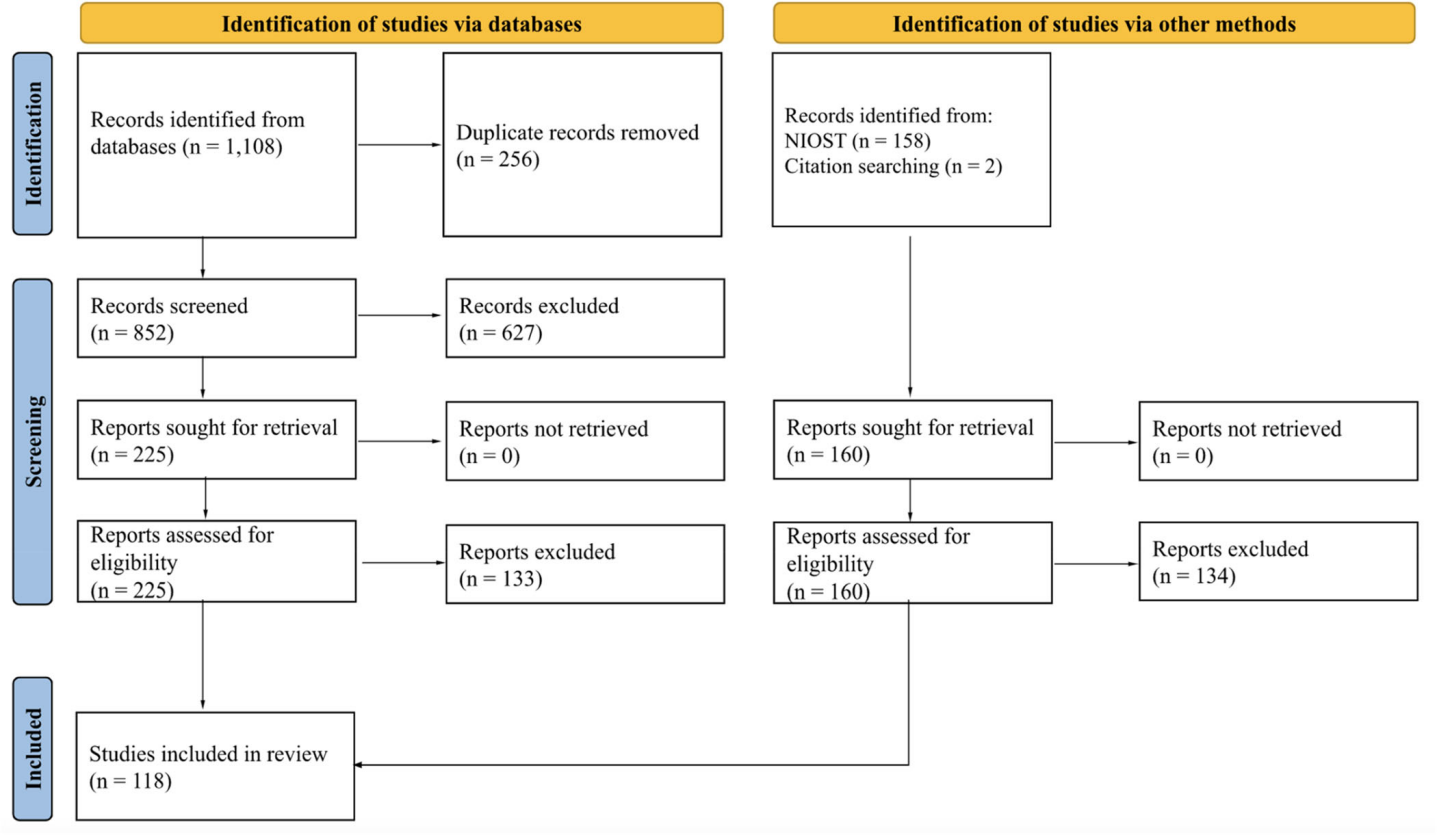
PRISMA Flowchart.

Of the 1108 original database articles, 256 duplicates were removed, leaving 852 original articles. These articles were screened by the first author, who consulted with the second author to reach agreement in any cases of uncertainty. We excluded 627 articles via abstract screening using the inclusion/exclusion criteria. Of the remaining 225 articles, we excluded 133 via full‐text screening, leaving 92 articles. Of the 158 original NIOST articles, we excluded 134, leaving 24 NIOST articles. We later identified two additional articles that qualified to be part of the sample, for a total of 118 included articles.^2^ See Figure 1 for the PRISMA flowchart.

## 5 Results

### 5.1 Study Features

Of the included articles, the majority (*n* = 106, 90%) were published in peer‐reviewed journals, 9 (8%) were reports, and 3 (3%) were working papers. About one‐third used quantitative methods (*n* = 42, 36%), qualitative methods (*n* = 37, 31%), or mixed methods (*n* = 39, 33%), respectively. Studies were primarily nonexperimental (*n* = 94, 80%) as opposed to quasi‐experimental (*n* = 16, 14%) or experimental (*n* = 8, 7%). Forty‐seven articles (40%) explicitly identified one or more theoretical or conceptual framework in their study. The most common framework was PYD (*n* = 9, 8%), followed by social justice/critical theories (*n* = 6, 5%) and culturally responsive pedagogy (*n* = 5, 4%). See Table 1 for the study features of included studies.

**Table 1:** Study features of included studies, *N* = 118.

Study feature	*n* (%)
Types of publications	
Peer‐reviewed journals	106 (90%)
Reports	9 (8%)
Working papers	3 (3%)
Research methods used	
Quantitative	42 (36%)
Qualitative	37 (31%)
Mixed	39 (33%)
Types of research designs	
Non‐experimental	94 (80%)
Quasi‐experimental	16 (14%)
Experimental	8 (7%)
Theoretical/conceptual framework used	47 (40%)
Positive youth development	9 (8%)
Social justice/critical theory	6 (5%)
Culturally responsive pedagogy	5 (4%)
Social capital theory	4 (3%)
Ecological systems theory	4 (3%)
Self‐determination theory	4 (3%)
Constructivist/experiential learning theory	3 (3%)
Sociocultural theory	3 (3%)
Social cognitive theory	3 (3%)
Asset theory, funds of knowledge	3 (3%)
Empowerment theory	1 (< 1%)
Theory of planned behavior	1 (< 1%)
Hierarchical model of self‐esteem	1 (< 1%)
Social action theory	1 (< 1%)
Maslow's hierarchy of needs	1 (< 1%)
Population‐based health promotion model	1 (< 1%)
Standpoint theory	1 (< 1%)
Flow and by stage‐environment fit theory	1 (< 1%)

The majority of studies analyzed data from the youth participants themselves, whether via surveys, interviews, and/or focus groups (*n* = 88, 75%). The most common data source used in the studies was youth participant surveys (*n* = 64, 54%), followed by researcher observations/field notes (*n* = 39, 33%) and youth participant interviews (*n* = 35, 30%). One‐quarter of studies analyzed data from OST non‐leadership staff (*n* = 29, 25%), while nine studies (8%) analyzed data from OST leaders like program directors. Thirteen studies (11%) analyzed data collected from parents or guardians, using interviews (*n* = 8, 7%), surveys (*n* = 5, 4%), and/or focus groups (*n* = 1, < 1%). Twenty‐two studies (19%) relied on program‐related documents or artifacts as data. See Table 2 for the data sources of included studies.

**Table 2:** Data sources of included studies, *N* = 118.

Data source	*n* (%)
Youth participant data (any method)	88 (75%)
Participant surveys	64 (54%)
Participant interviews	35 (30%)
Participant focus groups	17 (14%)
Researcher observations/field notes	39 (33%)
OST non‐leadership staff (any method)	29 (25%)
OST non‐leadership staff interviews	22 (19%)
OST non‐leadership staff surveys	10 (8%)
OST non‐leadership staff focus groups	6 (5%)
Program documents/artifacts	22 (19%)
School records (any method)	16 (14%)
Standardized test scores	10 (8%)
Attendance	9 (8%)
Graduation rates	4 (3%)
Grades	3 (3%)
Conduct/behavior	1 (< 1%)
Suspension rates	1 (< 1%)
Parent/guardian (any method)	13 (11%)
Parent/guardian interviews	8 (7%)
Parent/guardian surveys	5 (4%)
Parent/guardian focus groups	1 (< 1%)
OST leadership (any method)	9 (8%)
OST leadership interviews	8 (7%)
OST leadership surveys	2 (2%)
OST leadership focus groups	1 (< 1%)
School staff surveys or interviews	3 (3%)
Physical health indicators	3 (3%)
Police department arrest records	1 (< 1%)
National experts in OST research, policy and practice	1 (< 1%)

We also coded study outcomes. Studies examined the impact of OST programs on a variety of student outcomes, most often reporting positive effects, including: youth intrapersonal social‐emotional development (e.g., self‐awareness, confidence) (*n* = 21, 18%); interpersonal social‐emotional development (e.g., teamwork, social skills) (*n* = 19, 16%); STEM or literacy skills or knowledge (*n* = 16, 14%); college or graduate school aspiration, application, acceptance or enrollment (*n* = 16, 14%); STEM interest, attitudes or identity (*n* = 15, 13%); academic achievement (i.e., GPA or test scores) (*n* = 12, 10%); professional development or career readiness (*n* = 12, 10%); civic values or behaviors (*n* = 9, 8%); school attendance (*n* = 9, 8%); mental health symptoms (*n* = 7, 6%); high school graduation (*n* = 7, 6%); physical health (*n* = 5, 4%); cognitive development (*n* = 3, 3%); risk behaviors, skills or attitudes (e.g., crime or substance use attitudes) (*n* = 3, 3%); academic motivation or self‐efficacy (*n* = 3, 3%); school suspension rates or in‐school behavior (*n* = 2, 2%). Very few studies disaggregated student data to be able to draw conclusions about the impact of the program on subgroups of youth.

Three studies assessed outcomes related to parents: two examined parent involvement and one examined parent mental health. A number of studies (*n* = 67) assessed one or more factors related to program implementation, such as program fidelity, dosage, or participant engagement. Finally, nine studies did not focus on one particular program, but instead drew evidence of OST best practices from a broad pool of OST program attendees, parents, or staff. See Table 3 for the study outcomes of included studies.

**Table 3:** Study outcomes of included studies, *N* = 118.

Outcomes	*n* (%)
Youth outcomes	74 (63%)
Intrapersonal social‐emotional development	21 (18%)
Interpersonal social‐emotional development	19 (16%)
STEM or literacy skills/knowledge	16 (14%)
College or graduate school aspiration, application, acceptance or enrollment	16 (14%)
STEM interest, attitudes or identity	15 (13%)
Academic achievement	12 (10%)
Professional development/career readiness	12 (10%)
Civic values or behaviors	9 (8%)
School attendance	9 (8%)
Mental health symptoms	7 (6%)
High school graduation	7 (6%)
Physical health	5 (4%)
Cognitive development	3 (3%)
Risk behaviors, skills, or attitudes	3 (3%)
Academic motivation/self‐efficacy	3 (3%)
School‐based behavior measures (e.g., school suspension)	2 (2%)
Parent outcomes	3 (3%)
Implementation outcomes	67 (57%)
Participant engagement/satisfaction	47 (40%)
General success/challenges	35 (30%)
Staffing/training	12 (10%)
Recruitment efforts	8 (7%)

### 5.2 Program and Participant Features

Across all included articles, we investigated a total of 100 discrete OST programs. Programs were distributed widely across the U.S., covering the mid‐Atlantic (*n* = 25 programs, 25%), Midwest (*n* = 17, 17%), West (*n* = 13, 13%), Northeast (*n* = 11, 11%), Southeast (*n* = 10, 10%), and Southwest (*n* = 8, 8%) regions. Ten programs (10%) did not report location in terms of U.S. state or region. Six programs (6%) were in multiple regions. Programs were considered *urban* if the article reported that they were located in a major city/metropolitan area. Of the 100 OST programs, 29 (29%) did not describe their setting. Of the remaining 71 programs, the majority (*n* = 57, 80%) were in urban settings; four (6%) were in suburban settings; and four (6%) were in rural settings. An additional three programs (4%) served youth across multiple settings, and three programs (4%) were delivered online due to the COVID‐19 pandemic. Of the 100 programs, 31 (*n* = 31%) were delivered only during the summer months. The remaining programs (*n* = 69, 69%) were delivered either during the school year or year round.

Specific grade level or age of participants was not reported for three programs (3%). Of the remaining 97 programs, slightly more OST programs targeted HS students, or youth aged 14 to 18 (*n* = 45, 46%) compared to middle school students, or youth aged 11–13 years old (*n* = 37, 38%). Fifteen programs (15%) served both MS and HS students.

The gender of program participants was coded into three categories: a) gender statistics not reported; b) mixed gender; or c) single gender. For nearly half of the 100 programs (*n* = 44, 44%), participant gender statistics were not reported. Of the remaining 56 programs that did report gender, most programs were mixed gender (*n* = 39, 70%); 13 programs (23%) were girls‐only; and four programs (7%) were boys‐only. Of note, only one study recognized any gender outside of the binary: in a study of Project SEED (Summer Experiences for the Economically Disadvantaged), < 1% of the participants identified as nonbinary (Nadelson et al. 2022).

The race/ethnicity of participants was coded into three categories: a) race/ethnicity statistics not reported; b) diverse (i.e., no particular racial/ethnic group constituted 60% or more of the OST program participants); or c) predominantly one racial/ethnic group (i.e., one group comprised 60% or more of the OST program participants). Participant race/ethnicity statistics were not reported for more than half of the programs (*n* = 54, 54%). Of the remaining 46 programs that did report participant race/ethnicity, 18 programs (39%) were diverse; 17 programs (37%) had majority Black or African American participants; 9 programs (20%) had majority Latinx participants; one program (2%) had majority Hmong participants; and one program (2%) had majority white participants. Three programs (*n* = 7%) reported serving any Indigenous participants, with percentages of Indigenous youth ranging from 1% to 17%. See Table 4 for the program features of including studies.

**Table 4:** Program and participant features of included programs, *N* = 100.

Program/Participant feature	*n* (%)
Region	
Mid‐Atlantic	25 (25%)
Midwest	17 (17%)
West	13 (13%)
Northeast	11 (11%)
Southeast	10 (10%)
Region not reported	10 (10%)
Southwest	8 (8%)
Multiple regions	6 (6%)
Setting	
Urban	57 (57%)
Setting not reported	29 (29%)
Suburban	4 (4%)
Rural	4 (4%)
Multiple settings	3 (3%)
Online	3 (3%)
Timing	
After school only	50 (50%)
Summer only	32 (32%)
After school and summer	18 (18%)
Participant grade level	
High school only	45 (45%)
Middle school only	37 (37%)
Mix of middle and high school	15 (15%)
Not reported	3 (3%)
Participant gender	
Not reported	44 (44%)
Mixed gender	39 (39%)
Girls only	13 (13%)
Boys only	4 (4%)
Participant race/ethnicity	
Not reported	54 (54%)
Diverse	18 (18%)
Majority Black/African American	17 (17%)
Majority Latinx	9 (9%)
Majority Hmong	1 (1%)
Majority white	1 (1%)

### 5.3 Staffing

Staffing is a critical part of any OST program. As the literature has consistently concluded, relationships between staff and youth participants can promote or hinder program success (Greene et al. 2013). Participants expressed that OST staff felt more like family members (e.g., “big sisters”) or “friends” in the more informal OST atmosphere, in contrast to stricter relationships with teachers at school (Markowitz 2012). As such, OST staff have a unique opportunity to make a substantial impact in youth's lives.

#### 5.3.1 Staff Qualities

The OST programs in this review were run by various people, including hired staff, community volunteers, AmeriCorps members, teachers, artists, business professionals, librarians, park staff, scientists, researchers, undergraduate students, and graduate students. In a few cases, peers or near‐peers delivered programming. In the Young People's Project (Tucker‐Raymond et al. 2016), college students trained HS students to teach math and computer science to younger students. Another program, Better Futures Project (Geenen et al. 2015), designed to promote post‐secondary education for foster HS youth experiencing a mental health condition, hired peer coaches (under 28 years old) with shared experiences around foster care and/or mental health.

Having staff who shared lived experiences with the participants was a powerful approach, especially in mentorship‐based OST programs. In one study with adolescent Black girls and their mentors, all mentors and the majority of the girls expressed that mentors should share their mentees’ racial identity. According to one participant, “It's very nice to have somebody that's going through something that you're going to be going through in the future and tell you about the situation they're having. We relate” (Kayser et al. 2018, p. 48). Meanwhile, staff members of Educational Excellence (EE) (Baldridge 2018), many of whom grew up in the same communities as participants, were strong and caring advocates, using their knowledge and experiences to help youth navigate various challenges. For example, staff guided youth and families to schools that were seen as affirming spaces with rigorous and culturally relevant opportunities. Staff also monitored students’ report cards and test scores, enabling them to intervene as necessary, and mediated hardships within families as appropriate. Importantly, the staff at EE helped students learn how to advocate for themselves as well.

When full‐time staff members do not share similar backgrounds with the participants, programs can invite community members to lead workshops, and presentations or otherwise be involved in a less time‐intensive way. For example, Techbridge Girls invited parents, siblings, and members of professional groups (e.g., the National Society of Black Engineers) to share their knowledge with program participants, recognizing “how important it is for youth to see women, people of color, individuals with disabilities, immigrants, and people from other underrepresented groups working in STEM fields” (Kekelis et al. 2017, p. 15).

However, a few programs presented counterexamples in which OST staff and participants did not share similar backgrounds. In these cases, it was important for OST staff to demonstrate openness and collaboration, along with a pedagogical approach aligned with program goals. For example, a white teacher at an afterschool literacy program for Latina HS students believed that she was able to develop trusting relationships with the girls, noting, “When I feel most synchronized with the girls is when they are genuinely owning it [their writing]” (García and Gaddes 2012, p. 149). She intentionally facilitated a space that invited youths’ authentic voices.

#### 5.3.2 Staff Training

Staff training covered both content knowledge (e.g., adolescent development) and techniques (e.g., culturally responsive teaching, working with parents, and mentoring). Training was conducted by OST leadership, local experts (e.g., social workers), or national organizations. In one case, undergraduate students took a 2‐credit course in the spring that allowed them to learn about the summer OST program, the community, and mentoring, in preparation for their work (Rogers et al. 2020). Oftentimes, training was conducted before staffs’ start date and continued throughout the program to reinforce certain topics and provide additional support as needs arose. Feedback from staff also helped shape the program and future trainings.

One challenge reported in a few studies was helping OST staff find the right balance between being directive and promoting youth autonomy. As such, it can be helpful for program leaders to be explicit about the role and expectations of staff members, teach relevant techniques, practice scenarios, and incorporate activities within the curriculum to help staff achieve the appropriate balance. In the case of Techbridge Girls, for example, OST staff at first were trained on subject matter skills so that mentors would be able to help girls troubleshoot the technology. However, based on their observations, “After that first year, we realized that the mentor training needed to focus less on technology and more on the practice of mentoring: how to support projects without taking them over” (Kekelis et al. 2017, p. 10). In the case of the Youth Engaged in Leadership and Learning curriculum delivered to adolescents in public housing, the program manual identified the role of staff (“facilitator, mentor, and partner”) as well as how to build in youth voice (e.g., facilitating community circles) (Anyon et al. 2018).

#### 5.3.3 Staff Retention and Recruitment

Staff turnover is an issue in most OST programs. To the participants, turnover can feel like “losing a family member” (McGuiness‐Carmichael 2019), leaving youth feeling abandoned or discouraged. A disruption in trust between youth and the program can be challenging for new staff to rebuild, unsettling program stability. To mitigate the harmful effects of staff turnover, recommendations from youth and staff include maintaining program consistency, communication (e.g., providing notice about staff transitions), and relationship building (e.g., introducing participants to new staff along with staff they already know, and offering intentional time for participants and new staff to get to know each other) (McGuiness‐Carmichael 2019).

Staff recruitment can be challenging for a variety of reasons, including commute time, safety of the neighborhood, and competing options and priorities (Garcia et al. 2020). OST leaders play an important role in staff retention and overall success of the program. At Higher Achievement, researchers found that the centers that struggled to recruit mentors to their program also had the most turnover in leadership, concluding that “experienced leaders may be better recruiters, and mentors continue to volunteer year after year, in part because of their relationships with center leaders” (Garcia et al. 2020, p. 15). Researchers of the program Sport Hartford determined that staff commitment enabled the program to build and maintain trust with the community. As one participant's mother explained, “You've always been there, no matter what… you know, you've never given up on the kids” (Bruening et al. 2015, p. 97).

One approach is for programs to hire program alumni. This serves a number of purposes. First, it can promote youth leadership development by providing a pathway for participants to stay involved with increasing responsibility. Second, it can be a strategic hiring option for programs that may otherwise have trouble recruiting staff. Third, hiring alumni can increase the number of staff who live in or come from the communities served by the program (Pyne et al. 2020). Intermediary positions, such as teaching assistants or counselors‐in‐training, can also help keep the staff to participant ratio low while providing near‐peer mentorship. Even without formalized roles, participants who return to a program year after year play an important role in mentoring new participants and fostering an inclusive environment (Burch et al. 2019).

### 5.4 Participant Recruitment

When Jones and Jones (2020) asked participants what attracted them to the Youth Enrichment Services summer program, they realized, “The attracting factors basically are forms of capital: financial, human, and social” (p. 65). This held true across a number of programs. Youth were interested in programs that offered opportunities to learn new skills or expand their skillset (Laurenzano et al. 2021), earn money (Jones and Jones 2020; Laurenzano et al. 2021), and spend time with friends or make new friends (Jones and Jones 2020). Whalen et al. (2016) expanded on how social affiliation–being a member of a group and building relationships with like‐minded peers and adult leaders–drew adolescents to an afterschool physical activity club.

Youth were also drawn to programs because of an existing relationship with an adult, family member, or friend who recommended or encouraged attendance. For example, most of the participants in Project SEED had joined because they were encouraged to apply by a HS chemistry teacher familiar with the program, and the next most popular method was word of mouth from a family member or friend (Nadelson et al. 2022). For school‐site programs, OST staff who also work at the school during the day have a unique opportunity to recruit students. Students cited “calling students down on the announcements”, “talking to us in the hallways” and having “received a personal invitation” as reasons why they attended club sessions (Whalen et al. 2016, p. 644). One student even mentioned that the staff member would “call home and ask a mum if the student can come to the club” (Whalen et al. 2016, p. 644). These behaviors were interpreted by students as positive rather than an unwanted intrusion.

Youth Arts Initiative (YAI), hosted by the Boys & Girls Clubs of America (BGCA), used a number of tactics to attract participants to its art classes (Hartmann and McClanahan 2020). Each instructor, many of whom were practicing professional artists, was hired 1 month before their class began so that they could visit the Clubs, build relationships with youth and encourage them to try out the class. Youth were attracted to the experience and enthusiasm of the artists. The art studios with near‐professional equipment also garnered attention and helped word spread. When their peers saw the artwork hanging in the space or watched the culminating dance performance on YouTube, as one participant explained, “It motivates them to think… “Hey, this is something I might be able to do.” It gets them thinking it might be fun for them” (Hartmann and McClanahan 2020, p. 16). YAI also sought youth input via surveys and informal conversations, which helped them continue to align programming to youth's interests. YAI participants showed higher attendance and engagement than Club members who did not attend YAI classes.

Other programs were intentionally designed to remove barriers by hosting programming in places where youth already spent their time, including school buildings and other community or neighborhood sites. For example, NeighborWorks America offered afterschool programming within affordable housing communities, which resolved transportation issues like cost, travel time, and safety (Montoya 2020). In another example, the program 4 Youth, By Youth was initiated by the Baltimore County Public Libraries when they noticed an increase in adolescent visitors after school (Fields and Rafferty 2012). The library partnered with 4‐H to provide programming related to workforce readiness, science, nutrition, and community engagement and leadership during afterschool hours. Finally, some programs attracted participants in low‐income areas where there were few safe, structured afterschool activities available, such as two cases of park‐based programs designed for middle schoolers in urban neighborhoods experiencing high levels of violence (Frazier et al. 2015; Goodman et al. 2021). In this way, programs were delivered strategically in ways and places that were accessible, safe, and desirable to youth.

### 5.5 Participant Engagement

Reasons why a student is initially drawn to a program may not be the same ones that sustain participation (Jones and Jones 2020). This review yielded seven engagement strategies: agency, relevance, contribution, competence, belonging, exposure, and safety/wellness. Programs that involved *agency* facilitated ways for youth to have choices and make decisions in the programming. Programs that use strategies to make activities applicable to youths’ interests and life experiences promote *relevance*. We use the term *contribution* to signify activities that spur youths’ consciousness as participants and leaders in their communities. *Competence* is indicative of efforts to increase task complexity, including leadership roles, with scaffolding. Programs cultivated *belonging* when they prioritized relationship building, group bonding, celebrations, and rituals. *Exposure* included going on field trips or having youth engage in other novel experiences. Finally, *safety/wellness* included activities to support physical safety as well as wellness‐related strategies like access to food and connecting youth to resources. See Table 5 for the list of engagement strategies with examples.

**Table 5:** Engagement strategies.

Dimension	Recommendations	Examples
Agency	Allow choice and autonomy Invite youth to be decision‐makers Solicit and incorporate participant feedback	The program director at Educational Excellence asks students, “You know we're going to do a math class, you know we're going to do an English class. Is there anything you might want to learn about?” (Baldridge 2018, p. 13) Holstead et al. (2015) recommend formally involving youth in programming by collecting feedback, creating youth advisory boards, or inviting youth to help with hiring decisions “Mostly teenagers basically don't have any say, and people overlook them a lot. But in PeaceJam you're the main people, and teenagers are, like, controlling it” (Jones et al. 2013, p. 14)
Relevance	Applicable to real life Build upon youth interests and experiences Explicitly explain what transferable skills are and how skills developed in the program will help them succeed Incorporate hands‐on learning	One participant met “important people from the government” through the program Greening Western Queens, and said that she kept their contact information with the intent to apply for an internship in the future (De Jesús et al. 2015, p. 24)A science lesson for ELLs involved introducing a new word (“aquaculture”), measuring water temperature in a nearby pond, testing the water samples in the lab, and then visiting a local water treatment plant (Matthews and Mellom 2012)
Contribution	Invite students to reflect on and take action related to social justice in their communities and the world Increase participants’ awareness of their roles within their communities	A high schooler who taught math to younger students in the Young People's Project said, “I feel like giving back to the community, to be able to give them something they didn't have–like the older people in the town look down on us and say that's something that they didn't have” (Tucker‐Raymond et al. 2016, p. 1032) A journal prompt for middle school boys in the REACH Harlem afterschool basketball program was, “How have you been a role model in your community this week?” (Marttinen et al. 2019)
Competence	Offer opportunities for challenge Set high expectations Provide scaffolding and support Create pathways for participants to take on more leadership as they progress in a program	For participants of PeaceJammers, the feeling of success in accomplishing a goal led to a positive experience of the program, which in turn led to increased participation (Jones et al. 2013) Youth were invited to be junior counselors‐in‐training during their park's summer camp if they attended a minimum number of sessions and/or demonstrated competence in core skills (Frazier et al. 2015) Educational Excellence used asset‐rich language (“scholars”, “college‐bound”), both in conversation and physically represented in classrooms, offices, and program documents (Baldridge 2018)
Belonging	Set structures and practices to build relationships among staff and participants Celebrate cultural and linguistic assets Create traditions and rituals Invite friends, family, and other community members to take part in activities or events	At New Urban Arts (Ustach 2020), staff greeted youth at the door by name every day Physical activity clubs ordered t‐shirts and developed club mantras to promote group unity (Whalen et al. 2016) Youth Arts Initiative staff at BGCA (Hartmann and McClanahan 2020) texted, emailed, and posted on social media to update parents and share photos and videos of youth work Participants in a STEM summer program for ELL students created year‐round youth networks for ongoing support (Matthews and Mellom 2012)
Exposure	Expose participants to new ideas, activities, places, or people Consider offering field trips to facilitate community awareness and connections	The most meaningful parts of the YMCA Youth Institute were community service and travel opportunities, “which allowed [participants] to see beyond their neighborhoods” (O'Donnell and Kirkner 2016, p. 21) One objective of a summer program for newcomers was for participants to learn about their community and available resources (Symons and Ponzio 2019)
Safety & Wellness	Promote physical safety both at the program and during transportation to and from Consider offering funding or stipends Provide food or snacks Connect youth to other resources in the community as needed	NeighborWorks America staff walked youth from the bus stop to the program (Montoya 2020) Project Step‐Up (Greene et al. 2013) hosted afterschool academic and social‐emotional group sessions infused with features of typical mental health treatment like counseling, family intervention, and referrals. At an afterschool program aimed at reducing violence‐related behaviors (Risisky et al. 2019), staff were trained in mediation skills to help participants resolve conflict

In practice, these engagement strategies are interrelated and are facilitated by various pedagogical and curricular decisions. Below, we outline four recommendations derived from the literature: culturally responsive and asset‐based practices, deliberate attendance policies, project‐based learning, and a culminating showcase or event.

#### 5.5.1 Culturally Responsive and Asset‐Based Practices

Various programs took a culturally responsive, assets‐based approach, intentionally integrating participants’ cultural knowledge into curriculum and pedagogy. For example, an Upward Bound Summer Program used a culturally relevant science curriculum (Garvin‐Hudson and Jackson 2018) to foster Black HS students’ science interest and identity. In one chemistry unit, students donated locks of hair for chemical testing to learn about chemical relaxers and the properties of acids and bases. According to interviews, students were particularly inspired and encouraged by the weekly Lab out Loud initiative, during which they interacted with Black professionals in STEM careers. In another example, teachers in the Newcomer English Language Learner Enrichment Academy (López et al. 2020) worked closely with the local refugee resettlement center to learn about their students’ cultures and experiences. One staff member explained, “It's a lot of fun having conversations and trying to learn about their culture, and… having them teach me something new… Building that relationship and rapport makes it so much more fun to teach” (López et al. 2020, p. 95).

#### 5.5.2 Deliberate Attendance Policies

A number of studies described the importance of implementing attendance policies that aligned with program goals (e.g., skill‐building, exposure, creativity) while meeting the needs of participants. For example, for the skill‐building classes offered by the Youth Arts Initiative at BGCA (Hartmann and McClanahan 2020), participants were required to attend regularly and stay for the entire class (typically 2 h) to meet the goal of learning the skill. Staff sent letters home and spoke with parents to explain the importance of class attendance. A few staff created contracts or held parent meetings to reinforce the attendance commitment.

Other programs offered flexible attendance policies. The goal of Higher Achievement, an academic program for middle school “scholars,” was “to help young people develop skills, behaviors, and attitudes that will improve their academic performance and ultimately increase their acceptance in competitive high schools that could launch them into college and careers” (Garcia et al. 2020, p. 3). Scholars were expected to attend the program 3 days a week (after school) or 5 days a week (during the summer). However, centers allowed exceptions to avoid scholar drop‐out, such as missing parts of the program to attend to other commitments or responsibilities, and allowed scholars with “poor” attendance to stay on the roster.

Finally, in other cases, drop‐in attendance policies were most appropriate. The nonprofit New Urban Arts (Ustach 2020) ran the largest afterschool arts program for HS students in Rhode Island. The goal of the drop‐in art studio was that youth “make a permanent place for creativity and imagination in their lives” (Ustach 2020, p. 4). New Urban Arts fostered community and relationships while allowing youth to choose which days to stop in, what projects to work on, and how long they wanted to stay. In surveys, participants reflected on the benefits of this policy: “Being told something is mandated might make me feel anchored down and not want to attend. Personally, it was such a place that embodied freedom compared to everything else”; and “With the many commitments that I had at the time, it would have been difficult to also add fixed blocks of time into my schedule” (Ustach 2020, p. 8).

#### 5.5.3 Project‐Based Learning

Reviewed programs often featured project‐based learning (PBL), a method by which youth work in groups to solve authentic, challenging problems. PBL facilitated agency, relevance, and competence, as well as critical thinking and collaboration. Many OST programs invited participants to work on a real‐world project that was meaningful to them and their communities. For example, as part of Beyond Blackboards (Blanchard et al. 2015), an afterschool engineering and robotics club in rural Texas middle schools, teams of participants designed LEGO robots to accomplish “missions” based on pressing societal concerns identified by the National Academy of Engineering 21st Century Grand Challenges. One year, participants combined LEGOs with solar panels, wind turbines, and power meters to explore solar and wind power generation. According to Blanchard et al. (2015), schools that hosted the afterschool club saw a school‐wide increase in interest in engineering careers, indicating a school culture shift.

#### 5.5.4 Culminating Event

Culminating events served many purposes: to enable participants to have a tangible product for their efforts; to provide motivation and a common goal to work toward; to celebrate; and to engage family, friends and community, and to provide an opportunity for participants to explain their work to them. Culminating events were incorporated in nearly all types of OST programs. Participants created posters for research conferences, held art shows or performances, delivered presentations to community members, and played in sports tournaments. In the case of a before‐school writing club for MS students, however, the culminating event was less valuable than expected (Ruben and Moll 2013). The original club flier advertised that the club participants would submit to and attend a statewide writing festival. Only 11 of the 16 participants attended the festival, and while they spoke positively about the experience, they noted that the festival did not drive club attendance or engagement, likely because these students were already highly intrinsically motivated to write. Considering participants’ sources of motivation and interest can help program developers design culminating events in meaningful ways.

#### 5.5.5 Disruptions to Engagement

Grant et al. (2016), in their qualitative investigation of ten Summer Youth Employment Program (SYEP) participants, revealed how youth who were the most marginalized experienced the most disconnection from the program. Of the ten youth interviewed, three were involved in the juvenile justice system, four were parents, one was pregnant, two were in foster care, and three had disabilities. Through ecomapping, researchers learned that participants tended to have stressful or disrupted relationships across many domains, including family. Then, at their SYEP work placements, disconnection arose from feeling self‐conscious, judged by colleagues, or not fitting in; an unchallenging work environment; lack of opportunity to connect with other adults (e.g., at a worksite taking care of young children); and lack of support or direction from supervisors. As the researchers concluded, “Programs aimed at disenfranchised youth will need to identify youth who may be more disconnected from society… to design a plan with additional supports” that fit the ecological needs of the individual (Grant et al. 2016, p. 14).

In another program, the High School Short‐Term Research Experience for Underrepresented Persons (Rivers et al. 2020), NIH‐funded coordinating centers matched low‐income, underrepresented HS students with faculty mentors close to home for a summer research experience. Most program elements were rated highly by students, except for one: mentor matching. One potential reason for the low satisfaction with mentor matching is that students were assigned research that didn't align with their interests. This example highlights the necessity of incorporating youth interests and choice into programming.

Finally, systemic issues related to the limited availability of resources can have downstream effects on youth engagement. For afterschool physical activity clubs at Midwest urban high schools, youth were often hungry and lacked energy to exercise at dismissal (Maljak et al. 2014). Though snacks were an effective incentive, snacks were often bought by club leaders with their own money, and the inability to provide snacks regularly led to lower attendance and early dismissals. Attendance and engagement were also impacted by transportation challenges like waiting at bus stops or walking home in the dark, conflicting bus schedules, needing to transfer buses multiple times to get home, and parent/guardian work schedules (Maljak et al. 2014).

## 6 Discussion

In this systematic review, we synthesized 118 studies on OST programs for low‐income MS/HS youth, with a focus on staffing, participant recruitment, and participant engagement. This systematic review addressed critical questions about OST programs for low‐income adolescents through analyzing patterns in the research literature and identifying meaningful recommendations for OST practice (Alexander 2020). It is important to consider, however, that many programs exist around the country that are not formally researched, or that are researched but do not make their way into the literature, and are therefore not included in this review. This is reflective of a broader equity issue because research tends to be costly in terms of financial resources and time. The findings presented in this review should take into account this limitation.

### 6.1 Research Considerations

The included studies represent a variety of methods (quantitative, qualitative, and mixed methods) and incorporated the voices of people of diverse backgrounds and experiences (youth, parents, OST staff and leadership, community members, and some school staff) across the United States. The review included some highly powered randomized controlled trials (e.g., Heller 2014), which help establish evidence for OST effectiveness. A number of studies presented findings from formative or process evaluations, which program staff then used to make iterative programmatic changes. While this can be a resource‐intensive process, formative evaluations are powerful sources of information and opportunities for continuous improvement.

As reported by other systematic reviews on OST programs (e.g., Lester et al. 2020), the studies included in this review display a lack of consistency regarding measurement and reporting. The field as a whole would benefit from a set of best practices for OST research, such as by using theoretical frameworks like PYD to ground the studies or by establishing standardized reporting structures, which can help researchers learn from and build on each other's work while pushing the field forward in service of improving program quality and participant outcomes. For example, for the 100 programs reviewed, participant race/ethnicity and gender statistics were missing for about half of the programs (54% and 44%, respectively). These issues make it challenging to interpret or generalize study results, or to compare quality across programs. It is especially important for future research to report more detailed demographic information. In order for the field to generate meta‐analyses and draw empirical generalizations for OST, researchers must be able to rely on demographic data to identify individual and group differences. Without this information, we cannot know for whom OST programs work, to what extent, and how we can improve OST programs to better serve various subgroups of youth.

### 6.2 Program Considerations

Programs across the research literature provided a variety of opportunities to promote academics and PYD, aligning with youth's developmental stage and drawing on resources in the community. However, staff turnover plagues many OST initiatives. One recommendation gleaned from these articles is to establish pathways for youth to become volunteers or staff within a program, thereby replenishing staff with people who know the program and community well while maintaining and deepening the connection that had been established between a young person and the program. Another recommendation is to establish and support strong program leadership. Leaders can increase staff retention through building relationships and developing effective practices for staff recruitment. Importantly, professional development should not only teach required content knowledge but also facilitate learning soft skills like mentoring and how to talk to parents. When staff shared lived experiences with participants, they were able to connect, mentor, guide, and advocate for participants in particularly effective ways.

Findings suggest that effective strategies for identifying and recruiting low‐income MS/HS youth to OST programs include clearly communicating ways in which participation will increase capital, such as developing skills, building relationships, or earning money; relying on youth's existing relationships with friends, teachers, and counselors to generate interest and provide direct referrals; establishing partnerships with trusted organizations that serve the target population; and advertising participant products or activities online. Programs must attend to issues such as transportation, safety of the surrounding areas, and youth energy levels after a long school day to successfully recruit youth to a given program.

To promote engagement, OST programs encouraged youth agency, offered opportunities that were relevant to their lives, nurtured the development of competence, encouraged contribution to the larger community, exposed youth to new experiences, fostered a sense of belonging, and ensured safety and wellness. These engagement strategies have strong overlap with PYD competencies, specifically what are known as the “5 Cs”—competence, competence, character, caring, and connection (Lerner et al. 2005)—through building both internal and external assets (Benson et al. 2011). Programs expanded youth's social capital and access to resources by connecting youth with community members, strengthening ties with community‐based institutions, and explicitly teaching concrete skills like networking and employability competencies. Specific engagement approaches included culturally relevant pedagogy, deliberate attendance policies, project‐based learning, and culminating events. However, despite it being best practice to engage families in OST settings (NIOST 2006), programs included in this review generally did not involve families outside of recruitment activities and culminating events. OST programs should consider not only increasing family engagement opportunities, but also partnering with families and other community members to ensure that the program remains grounded in the values and principles of the community (NIOST 2006).

Findings suggest that youth disengage from programs when they are not able to develop positive relationships, lack clear purpose or direction, or are not offered activities that are meaningful or interesting to them. Youth who are the most marginalized, including youth contending with multiple stressors, benefit from OST staff who understand their needs and are able to provide additional support. Programs also incorporated trauma‐informed practices (TIP) in their programming, such as safety, trust, choice, collaboration, and empowerment (Smith 2019). TIP are important for this population because marginalized, low‐income youth are more likely to experience high chronic stress or trauma related to poverty, community violence, and/or social exclusion. When TIP are integrated into programming, OST spaces are better equipped to help youth regulate, engage, and build relationships.

Across all findings, repeated themes were relationships, safety, and trust. Successful programs were dependable community assets. Program staff served as confidants, mentors, advocates, and peer conflict mediators, developing family‐like relationships with youth. Through OST opportunities, communities came together to create safe spaces for youth to learn and grow.

## 7 Conclusion

This systematic review highlights the critical role OST programs can play in addressing inequities and fostering PYD for low‐income adolescents. By examining 118 studies representing 100 programs, we identify promising strategies for staffing, recruitment, and engagement that contribute to equitable access and meaningful participation. Programs prioritize strong leadership and professional development for staff, leverage culturally responsive and asset‐based practices, emphasize youth agency and relevance, and address systemic barriers such as cost and transportation to create inclusive environments that meet the needs of underserved youth. Policymakers, educators, and community leaders must continue to create safe, inclusive, and engaging environments that address the unique needs of low‐income youth.

Despite the promising practices identified, significant challenges remain, including systemic inequities in access, inconsistent program quality, and staff turnover. Addressing these barriers requires sustained investment and a commitment to justice‐oriented programming. Additionally, researchers must continue to play an important role in identifying evidence‐based OST practices through robust empirical studies and evaluations, delving into the mechanisms that drive program success and strategies to tailor programs to diverse populations and contexts. By continuing to build the evidence base and implementing equitable, evidence‐informed practices, OST programs can empower low‐income adolescents to thrive in their communities.

## Ethics Statement

The authors have nothing to report.

## Conflicts of Interest

The authors declare no conflicts of interest.

## Supporting information

Supplementary_materials.

## Data Availability

The authors have nothing to report.

## Endnotes

1 The Great Recession was a significant economic downturn—the most severe recession since World War II. Primarily triggered by the bursting of the U.S. housing bubble, it had widespread global effects, and recovery was prolonged.

2 References for all 118 included articles are listed in the Supplementary file.
