# Supplementary material for: Promising Practices of Out‐of‐School Time Programs for Low‐Income Adolescents: A Systematic Review
Source: J Adolesc. 2025 Apr 24;97(5):1145–60. doi: 10.1002/jad.12506 (PMC12217409; doi:10.1002/jad.12506)
Supplement: Supplementary file 1 — Supplementary_materials. [file JAD-97-1145-s001.docx]

**List of Articles Included in Review**

Anyon, Y., Kennedy, H., Durbahn, R., & Jenson, J. M. (2018). Youth-led participatory action research: Promoting youth voice and adult support in afterschool programs. *Afterschool Matters*, *27*, 10-18.

Avery, C. (2013). *Evaluation of the College Possible Program: Results from a randomized controlled trial*. *NBER Working Paper* 19562, National Bureau of Economic Research.

Baldridge, B. J. (2018). On educational advocacy and cultural work: Situating community-based youth work[ers] in broader educational discourse. *Teachers College Record*, *120*(2).

Bernier, A., & Fowler, R. H. (2020). Teens in a digital desert: Digital media literacy in an Arizona OST program. *Afterschool Matters*, *33*, 50-57.

Berry, T., & LaVelle, K. B. (2013). Comparing socioemotional outcomes for early adolescents who join after school for internal or external reasons. *The Journal of Early Adolescence*, *33*(1), 77–103. doi:[10.1177/0272431612466173](https://doi.org/10.1177/0272431612466173)

Blanchard, S., Judy, J., & Muller, C. (2015). Beyond Blackboards: Underserved middle school students in engineering. *Journal of Pre-College Engineering Education Research*, *5*(1). [doi:10.7771/2157-9288.1084](https://docs.lib.purdue.edu/jpeer/vol5/iss1/2/)

Boyer, K. A. M., & Tracz, S. M. (2014). Hmong high school students in afterschool: Effects on achievement, behavior, and self-esteem. *Afterschool Matters, 19*, 44-50.

Broder, E. D., Guilbert, K. E., Tinghitella, R. M., Murphy, S. M., Ghalambor, C. K., & Angeloni, L. M. (2019). Authentic science with dissemination increases self-efficacy of middle school students. *Integrative and Comparative Biology*, *59*(6), 1497-1508. [doi:10.1093/icb/icz140](https://academic.oup.com/icb/article/59/6/1497/5540331)

Bruening, J. E., Clark, B. S., & Mudrick, M. (2015). Sport-based youth development in practice: The long-term impacts of an urban after-school program for girls. *Journal of Park and Recreation Administration*, *33*(2), 87-103

Burch, D., Summer, D., Ward, E., Watt, C., & Feldman, D. (2019). Qualitative data from a mixed methods study of resilience in ENACT’s therapeutic theatre process Show UP! *Drama Therapy Review*, *5*(1), 117–138. [doi:10.1386/dtr.5.1.117_1](https://intellectdiscover.com/content/journals/10.1386/dtr.5.1.117_1)

Causey, K., Zuniga, M., Bailer, B., Ring, L., & Gil-Trejo, L. (2012). Using theater arts to engage Latino families in dialogue about adolescent sexual health: The PATH-AT program. *Journal of Health Care for the Poor and Underserved*, *23*(1), 347–357. [doi:10.1353/hpu.2012.0036](https://pubmed.ncbi.nlm.nih.gov/22643482/)

Chiappinelli, K. B., Moss, B. L., Lenz, D. S., Tonge, N. A., Joyce, A., Holt, G. E., Holt, L. E., & Woolsey, T. A. (2016). Evaluation to improve a high school summer science outreach program. *Journal of Microbiology & Biology Education*, *17*(2), 225–236. [doi:10.1128/jmbe.v17i2.1003](https://journals.asm.org/doi/full/10.1128/jmbe.v17i2.1003)

Crump, C., Ned, J., & Winkleby, M. A. (2015). The Stanford Medical Youth Science Program: Educational and science-related outcomes. *Advances in Health Sciences Education : Theory and Practice*, *20*(2), 457–466. [doi:10.1007/s10459-014-9540-6](https://pubmed.ncbi.nlm.nih.gov/25096792/)

Cummings, M., Whitlock, A., Draper, M., Renschler, L., Bastian, K., Cox, C. C., & Visker, J. D. (2013). “All Stars” for at-risk middle school students in an afterschool setting: A pilot program. *Journal of Substance Use*, *19*(6), 444–447. [doi:10.3109/14659891.2013.859751](https://www.tandfonline.com/doi/abs/10.3109/14659891.2013.859751)

Daniel, S. M., & Eley, C. (2018). Improving cohesion in our writing: Findings from an identity text workshop with resettled refugee teens. *Journal of Adolescent & Adult Literacy*, *61*(4), 421–431. [doi:10.1002/jaal.700](https://ila.onlinelibrary.wiley.com/doi/10.1002/jaal.700)

De Jesús, A., Oviedo, S., & Feliz, S. (2015). Global kids organizing in the global city: Generation of social capital in a youth organizing program. *Afterschool Matters*, *21*, 20-28.

Dwyer, E., & McCloskey, M. L. (2013). Literacy, teens, refugees, and soccer. *Refuge: Canada’s Journal on Refugees*, *29*(1), 87–101. [doi:10.25071/1920-7336.37509](https://refuge.journals.yorku.ca/index.php/refuge/article/view/37509)

Eyerman, S., & Hug, S. (2020). Balancing acts: Managing the tensions inherent in long-term youth-led projects. *Afterschool Matters*, *33*, 25-31.

Fashola, O. S. (2013). Evaluation of an extended school day program for African American males in the context of single gender schooling and schoolwide reform: A case for extending the school day for African American males. *Peabody Journal of Education*, *88*(4), 488–517. [doi:10.1080/0161956x.2013.821895](https://www.tandfonline.com/doi/full/10.1080/0161956X.2013.821895)

Fenzel, L. M., & Richardson, K. D. (2018). Use of out-of-school time with urban young adolescents: A critical component of successful NativityMiguel schools. *Educational Planning*, *25*(2), 25–32.

Fields, N. I., & Rafferty, E. (2012). Engaging library partners in 4-H programming. *Afterschool Matters, 15*, 26-31.

Frazier, S. L., Dinizulu, S. M., Rusch, D., Boustani, M. M., Mehta, T. G., & Reitz, K. (2015). Building resilience after school for early adolescents in urban poverty: Open trial of Leaders @ Play. *Administration and Policy in Mental Health and Mental Health Services Research*, *42*(6), 723–736. [doi:10.1007/s10488-014-0608-7](https://pubmed.ncbi.nlm.nih.gov/25425012/)

Friesem, Y., & Greene, K. (2020). Tuned in: The importance of peer feedback with foster youth creating media. *Reflective Practice*, *21*(5), 659–671. [doi:10.1080/14623943.2020.1798919](https://www.tandfonline.com/doi/full/10.1080/14623943.2020.1798919)

Frosini, C. (2017). An “I” in teen? Perceived agency in a youth development program. *Afterschool Matters*, *25*, 29-37.

Frost, L., Bovard, B., Bugarin, A., Johnson, B., Atha, M., Swanson, M., & Walsh-Haney, H. (2021). Continuing to meet the needs of middle school students in Southwest Florida despite COVID-19 restrictions. *Journal of STEM Outreach*, *4*(3).

Galoyan, T., Barany, A., Donaldson, J. P., Ward, N., & Hammrich, P. (2022). Connecting science, design thinking, and computational thinking through sports. *International Journal of Instruction*, *15*(1), 601–618.

García, A., & Gaddes, A. (2012). Weaving language and culture: Latina adolescent writers in an after-school writing project. *Reading & Writing Quarterly: Overcoming Learning Difficulties*, *28*(2), 143–163. [doi:10.1080/10573569.2012.651076](https://www.tandfonline.com/doi/full/10.1080/10573569.2012.651076)

Garcia, I., Grossman, J. B., Herrera, C., & Linden, L. L. (2020a). The impact of an intensive year-round middle school program on college attendance. *MDRC*, New York, NY

Garcia, I., Grossman, J. B., Herrera, C., Strassberger, M., Dixon, M., & Linden, L. (2020b). Aiming Higher: Assessing higher achievement’s out-of-school expansion efforts. *MDRC*, New York, NY

Garvin-Hudson, B., & Jackson, T. O. (2018). A case for culturally relevant science education in the summer for African American youth. *International Journal of Qualitative Studies in Education (QSE)*, *31*(8), 708–725. [doi:10.1080/09518398.2018.1478156](https://www.tandfonline.com/doi/full/10.1080/09518398.2018.1478156)

Geenen, S., Powers, L. E., Phillips, L. A., Nelson, M., McKenna, J., Winges-Yanez, N., Blanchette, L., Croskey, A., Dalton, L. D., Salazar, A., & Swank, P. (2015). Better futures: A randomized field test of a model for supporting young people in foster care with mental health challenges to participate in higher education. *The Journal of Behavioral Health Services & Research*, *42*(2), 150–171. [doi:10.1007/s11414-014-9451-6](https://link.springer.com/article/10.1007/s11414-014-9451-6)

Goodman, A. C., Ouellette, R. R., D’Agostino, E. M., Hansen, E., Lee, T., & Frazier, S. L. (2021). Promoting healthy trajectories for urban middle school youth through county‐funded, parks‐based after‐school programming. *Journal of Community Psychology*. [doi:10.1002/jcop.22587](https://onlinelibrary.wiley.com/doi/abs/10.1002/jcop.22587)

Gopalan, G., Alicea, S., Conover, K., Fuss, A., Gardner, L., Pardo, G., & McKay, M. (2013). Project Step-Up: Feasibility of a comprehensive school-based prevention program. *The Journal of Early Adolescence*, *33*(1), 131–154. [doi:10.1177/0272431612467536](https://journals.sagepub.com/doi/10.1177/0272431612467536)

Grant, N., Bennett, J., & Crawford, M. (2016). Evaluating the ecological impact of a youth program. *Journal of Youth Development*, *11*(3), 188-206

Greene, K. M., Lee, B., Constance, N., & Hynes, K. (2013). Examining youth and program predictors of engagement in out-of-school time programs. *Journal of Youth and Adolescence*, *42*(10), 1557–1572. [doi:10.1007/s10964-012-9814-3](https://pubmed.ncbi.nlm.nih.gov/22971849/)

Groome, M., & Rodríguez, L. M. (2014). How to build a robot: Collaborating to strengthen STEM programming in a citywide system. *Afterschool Matters*, *19*, 1-9.

Guthrie, J. F., & Cho, C. (2015). School and district-level characteristics associated with participation in the after-school snack component of the National School Lunch Program. *Journal of Hunger & Environmental Nutrition*, *10*(2), 176–188. doi:10.1080/19320248.2014.9627

Hartmann, T., & McClanahan, W. (2020). Designing for engagement: How high-quality arts OST programs can engage tweens. *Afterschool Matters*, *31*, 11-21.

Heinert, S., Kowalski, S., Quasim, N., Suarez, N., & Vanden Hoek, T. (2019). Empowering Chicago’s youths as the next generation of health advocates. *American Journal of Public Health*, *109*(7), 1025–1027. [doi:10.2105/AJPH.2019.305055](https://ajph.aphapublications.org/doi/10.2105/AJPH.2019.305055)

Heller, S. B. (2014). Summer jobs reduce violence among disadvantaged youth. *Science*, *346*(6214), 1219–1223. [doi:10.1126/science.1257809](https://www.science.org/doi/10.1126/science.1257809)

Herrera, C., Grossman, J. B., & Linden, L. L. (2013). *Staying on track: Testing Higher Achievement’s long-term impact on academic outcomes and high school choice*. A Public/Private Ventures Project Distributed by MDRC.

Hill, J. C., Lynne-Landsman, S. D., Graber, J. A., & Johnson, K. J. (2016). Evaluating a pregnancy and STI prevention programme in rural, at-risk, middle school girls in the USA. *Health Education Journal*, *75*(7), 882–894. [doi:10.1177/0017896916644845](https://journals.sagepub.com/doi/10.1177/0017896916644845)

Holstead, J., Hightower King, M., & Miller, A. (2015). Research-based practices in afterschool programs for high school youth. *Afterschool Matters*, *21*, 38-45.

Hynes, K.; Greene, K. M., & Constance, N. (2012). Helping youth prepare for careers: What can out-of-school time programs do? *Afterschool Matters*, *16*, 21-30.

Jones, D., & Jones, D. (2020). Transcend the summer slump: How summer programs can attract and retain low-income high school students. *Afterschool Matters*, 60–67.

Jones, J. N., Bench, J. H., Warnaar, B. L., & Stroup, J. T. (2013). Participation as relational process: Unpacking involvement in social action and community service. *Afterschool Matters*, *18*, 9-16.

Kabacoff, C., Srivastava, V., & Robinson, D. N. (2013). A summer academic research experience for disadvantaged youth. *CBE - Life Sciences Education*, *12*(3), 410–418. [doi:10.1187/cbe.12-12-0206](https://www.lifescied.org/doi/10.1187/cbe.12-12-0206)

Kayser, A. A., Jackson, A., & Kayser, B. (2018). A seat at the table: Listening to adolescent Black girls. *Afterschool Matters*, *28*, 44-49.

Kekelis, L., Ryoo, J. J., & McLeod, E. (2017). Making and mentors: What it takes to make them better together. *Afterschool Matters*, *26*, 8-17.

Kelly, C. (2012a). The cafeteria as contact zone: Developing a multicultural perspective through multilingual and multimodal literacies. *Journal of Adolescent & Adult Literacy*, *56*(4), 301–310. [doi:10.1002/jaal.00143](https://ila.onlinelibrary.wiley.com/doi/abs/10.1002/JAAL.00143)

Kelly, C. (2012b). Recognizing the "social" in literacy as a social practice: Building on the resources of nonmainstream students. *Journal of Adolescent & Adult Literacy*, *55*(7), 608-618

Kennedy, T. M., & Ceballo, R. (2013). Latino adolescents’ community violence exposure: After-school activities and *familismo* as risk and protective factors. *Social Development*, *22*(4), 663-682. [doi:10.1111/sode.12030](https://onlinelibrary.wiley.com/doi/10.1111/sode.12030)

King, K. M., Rice, J. A., Steinbock, S., Reno-Weber, B., Okpokho, I., Pile, A., & Carrico, K. (2015). Kentucky Teen Institute: Results of a 1-year, health advocacy training intervention for youth. *Health Promotion Practice*, *16*(6), 885–896. [doi:10.1177/1524839915588294](https://journals.sagepub.com/doi/10.1177/1524839915588294)

Koch, M., Gorges, T., & Penuel, W. R. (2012). Build IT: Scaling and sustaining an afterschool computer science program for girls. *Afterschool Matters*, *16*, 58-66.

Ladeji-Osias, J. O., Partlow, L. E., & Dillon, E. C. (2018). Using mobile application development and 3-D modeling to encourage minority male interest in computing and engineering. *IEEE Transactions on Education*, *61*(4), 274–280. [doi:10.1109/TE.2018.2826466](https://ieeexplore.ieee.org/document/8352000)

Laurenzano, M., Reilly, J. M., & Ross, S. M. (2021). *An evaluation of Dent Education’s Bet on Baltimore Summer Program—Year 3*. Center for Research and Reform in Education, Baltimore, MD

Laursen, S. L., Thiry, H., Archie, T., & Crane, R. (2013). Variations on a theme: Characteristics of out-of-school time science programs offered by distinct organization types. *Afterschool Matters*, *17*, 36-49.

Leos-Urbel, J. (2014). What is a summer job worth? The impact of summer youth employment on academic outcomes. *Journal of Policy Analysis and Management*, *33*(4), 891–911. [doi:10.1002/pam.21780](https://onlinelibrary.wiley.com/doi/abs/10.1002/pam.21780)

Leos-Urbel, J. (2015). What works after school? The relationship between after-school program quality, program attendance, and academic outcomes. *Youth & Society*, *47*(5), 684–706. [doi:10.1177/0044118x13513478](https://journals.sagepub.com/doi/10.1177/0044118X13513478)

Loeper, R. (2014). Combat sports bloggers, mad scientist poets, and comic scriptwriters: Engaging boys in writing on their own terms. *Afterschool Matters*, *19*, 36-43.

López, R. M., Lee, J. J., & Tung, R. (2020). Implementing a summer enrichment program for secondary newcomer students in a New England Community. *International Journal of Leadership in Education*, *23*(1), 77–92. [doi:10.1080/13603124.2019.1629629](https://www.tandfonline.com/doi/full/10.1080/13603124.2019.1629629)

Mac Iver, M. A., & Mac Iver, D. J. (2015). *The Baltimore City Schools Middle School*

*STEM Summer Program with VEX Robotics*. Baltimore Education Research Consortium

Mac Iver, M. A., & Mac Iver, D. J. (2019.) "STEMming" the swell of absenteeism in the middle years: Impacts of an urban district summer robotics program. *Urban Education*, *54*(1), 65-88

Maljak, K., Garn, A., McCaughtry, N., Kulik, N., Martin, J., Shen, B., Whalen, L., & Fahlman, M. (2014). Challenges in offering inner-city after-school physical activity clubs. *American Journal of Health Education*, *45*(5), 297–307. [doi:10.1080/19325037.2014.934414](https://www.tandfonline.com/doi/full/10.1080/19325037.2014.934414)

Markowitz, E. (2012). Exploring self-esteem in a girls’ sports program: Competencies and connections create change. *Afterschool Matters*, *16*, 11-20.

Martin, W., Gutierrez, J., & Muldoon, M. (2020). Digital badges forging connections between informal and higher education. *Afterschool Matters*, *33*, 16-24.

Marttinen, R., Johnston, K., Phillips, S., Fredrick, R. N., & Meza, B. (2019). REACH Harlem: Young urban boys’ experiences in an after-school PA positive youth development program. *Physical Education and Sport Pedagogy*, *24*(4), 373–389. [doi:10.1080/17408989.2019.1592147](https://www.tandfonline.com/doi/full/10.1080/17408989.2019.1592147)

Matthews, P. H., & Mellom, P. J. (2012). Shaping aspirations, awareness, academics, and action: Outcomes of summer enrichment programs for English-learning secondary students. *Journal of Advanced Academics*, *23*(2), 105–124. [doi:10.1177/1932202X12439197](https://journals.sagepub.com/doi/10.1177/1932202X12439197)

McGuiness-Carmichael, P. (2019). Youth perspectives on staff turnover in afterschool programs. *Afterschool Matters*, *30*, 19-23.

Monk, M. H., Baustian, M. M., Saari, C. R., Welsh, S., D’Elia, C. F., Powers, J. E., Gaston, S., & Francis, P. (2014). EnvironMentors: Mentoring at-risk high school students through university partnerships. *International Journal of Environmental and Science Education*, *9*(4), 385–397.

Monnat, S. M., Lounsbery, M. A. F., McKenzie, T. L., & Chandler, R. F. (2016). Associations between demographic characteristics and physical activity practices in Nevada schools. *Preventive Medicine*, *95*, S4–S9. [doi:10.1016/j.ypmed.2016.08.029](https://pubmed.ncbi.nlm.nih.gov/27565054/)

Montoya, M. A. (2020). Bringing afterschool home: OST programming in affordable housing communities. *Afterschool Matters*, *32*, 10-17.

Morgan, B., Gaitan, E., Polletta, V., Cheung, C., Aslan, L., Wolff, L, Cheung, V., Sassanfar, M., & Wallace, L. J. (2021). Adapting a hands-on youth development STEM program in the age of COVID-19: The LEAH Knox Scholars Program.” *Journal of STEM Outreach*, *4*(3), 1–6. [doi:10.15695/jstem/v4i3.08](https://www.jstemoutreach.org/article/27701-adapting-a-hands-on-youth-development-stem-program-in-the-age-of-covid-19-the-leah-knox-scholars-program/stats/all/pageviews)

Muno, A. (2014). And girl justice for all: Blending girl-specific & youth development practices. *Afterschool Matters*, *19*, 28-35.

Nadelson, L. S., Jemison, R. C., Soto, E., & Warner, D. L. (2022). Cultivating a new “SEED”: From an on-ground to online chemistry summer camp. *Journal of Chemical Education*, *99*(1), 129–139. [doi:10.1021/acs.jchemed.1c00280](https://pubs.acs.org/doi/10.1021/acs.jchemed.1c00280)

Nakamoto, J., & Rice, J. (2017). *Network for Teaching Entrepreneurship (NFTE) 2016 alumni survey findings.* WestEd, San Francisco, CA.

Noël, L. T., Rost, K., & Gromer, J. (2013). Depression prevention among rural preadolescent girls: A randomized controlled trial. *School Social Work Journal*, *38*(1), 1–18.

O’Donnell, J., & Kirkner, S. L. (2016). Helping low-income urban youth make the transition to early adulthood: A retrospective study of the YMCA Youth Institute. *Afterschool Matters*, *23*, 18–27.

Oparaji, J.-A. N., Nwachuku, E. L., & Rosenstock, J. M. (2015). Healthy minds: Promoting mental health for at-risk youths. *Psychiatric Services*, *66*(1), 104–105. [doi:10.1176/appi.ps.651008](https://ps.psychiatryonline.org/doi/10.1176/appi.ps.651008)

Papazian, A. E., Noam, G. G., Shah, A. M., & Rufo-McCormick, C. (2013). The quest for quality in afterschool science: The development and application of a new tool. *Afterschool Matters*, *18*, 17-24

Park, J. Y. (2016). “He didn’t add more evidence”: Using historical graphic novels to develop language learners’ disciplinary literacy. *Journal of Adolescent & Adult Literacy*, *60*(1), 35–43. [doi:10.1002/jaal.521](https://ila.onlinelibrary.wiley.com/doi/abs/10.1002/jaal.521)

Patton, C. L., Deutsch, N. L., & Das, A. (2016). Coordination, competition, and neutrality: Autonomy and relatedness patterns in girls’ interactions with mentors and peers. *Journal of Early Adolescence*, *36*(1), 29–53. [doi:10.1177/0272431614556349](https://journals.sagepub.com/doi/10.1177/0272431614556349)

Pavlakis, A. E. (2019). Creative youth development in the context of homelessness: Supporting stability while creating structural change. *Afterschool Matters*, *30*, 1-9.

Piazza, S. V., & Duncan, L. E. (2012). After-school literacy engagements with struggling readers. *Reading & Writing Quarterly*, *28*(3), 229–254

Pierce, B., Bowden, B., McCullagh, M., Diehl, A., Chissell, Z., Rodriguez, R., Berman, B. M., & D’Adamo, C. R. (2017). A summer health program for African-American high school students in Baltimore, Maryland: Community partnership for integrative health. *Explore: The Journal of Science And Healing*, *13*(3), 186–197. [doi:10.1016/j.explore.2017.02.002](https://pubmed.ncbi.nlm.nih.gov/28373062/)

Pyne, J., Messner, E., & Dee, T. S. (2020). *The dynamic effects of a summer learning program on behavioral engagement in school.* CEPA Working Paper No. 20-10, Stanford Center for Education Policy Analysis, Stanford, CA

Reynolds, R., & Chiu, M. M. (2013). Formal and informal context factors as contributors to student engagement in a guided discovery-based program of game design learning. *Learning, Media and Technology*, *38*(4), 429–462. [doi:10.1080/17439884.2013.779585](https://www.tandfonline.com/doi/full/10.1080/17439884.2013.779585)

Rieder, J., Moon, J.-Y., Joels, J., Shankar, V., Meissner, P., Johnson-Knox, E., Frohlich, B., Davies, S., & Wylie-Rosett, J. (2021). Trends in health behavior and weight outcomes following enhanced afterschool programming participation. *BMC Public Health*, *21*(672). [doi:10.1186/s12889-021-10700-4](https://bmcpublichealth.biomedcentral.com/articles/10.1186/s12889-021-10700-4)

Risisky, D., MacGregor, J., Smith, D., Abraham, J., & Archambault, M. (2019). Promoting pro-social skills to reduce violence among urban middle school youth. *Journal Of Youth Development*, *14*(4), 197–215. [doi:10.5195/jyd.2019.641](https://jyd.pitt.edu/ojs/jyd/article/view/19-14-04-PA-3)

Rivers, R., Norris, K. C., Hui, G., Halpern-Felsher, B., Dodge-Francis, C., Guerrero, L. R., Golshan, A., Brinkley, K., Tran, K., McLaughlin, S., Antolin, N., Yoshida, T., Caffey-Fleming, D. E., & Agodoa, L. (2020). The NIDDK High School Short-Term Research Experience for Underrepresented Persons. *Ethnicity & Disease*, *30*(1), 5–14. [doi:10.18865/ed.30.1.5 WE](https://pubmed.ncbi.nlm.nih.gov/31969778/)

Robbins, L. B., Ling, J., Toruner, E. K., Bourne, K. A., & Pfeiffer, K. A. (2016). Examining reach, dose, and fidelity of the “Girls on the Move” after-school physical activity club: A process evaluation. *BMC Public Health*, *16*(1). [doi:10.1186/s12889-016-3329-x](https://bmcpublichealth.biomedcentral.com/articles/10.1186/s12889-016-3329-x)

Robbins, L. B., Ling, J., & Wen, F. (2020). Attending after-school physical activity club 2 days a week attenuated an increase in percentage body fat and a decrease in fitness among adolescent girls at risk for obesity. *American Journal of Health Promotion*, *34*(5), 500–504. [doi:10.1177/0890117120915679](https://journals.sagepub.com/doi/abs/10.1177/0890117120915679)

Rogers, M., Livstrom, I., Roiger, B., & Smith, A. (2020). Growing North Minneapolis: Connecting youth and community through garden-based experiential learning. *Horttechnology*, *30*(1), 25–30. [doi:10.21273/HORTTECH04308-19](https://journals.ashs.org/horttech/view/journals/horttech/30/1/article-p25.xml)

Ruben, B., & Moll, L. (2013). Putting the heart back into writing: Nurturing voice in middle school students. *Middle School Journal*, *45*(2), 12–18.

Salto, L. M., Riggs, M. L., De Leon, D. D., Casiano, C. A., & De Leon, M. (2014). Underrepresented minority high school and college students report STEM-pipeline sustaining gains after participating in the Loma Linda University Summer Health Disparities Research Program. *PLOS ONE*, *9*(9:e108497). [doi:10.1371/journal.pone.0108497](https://journals.plos.org/plosone/article?id=10.1371/journal.pone.0108497)

Schwartz, A. E., Leos-Urbel, J., Silander, M., & Wiswall, M. (2014). *Making summer matter: The impact of youth employment on academic performance.* Working Paper #03-14, Institute for Education and Social Policy, New York, NY

Scott, K. A., & White, M. A. (2013). COMPUGIRLS’ standpoint: Culturally responsive computing and its effect on girls of color. *Urban Education*, *48*(5), 657–681. doi:[10.1177/0042085913491219](https://doi.org/10.1177/0042085913491219)

Stacki, S. (2012). Deepening understandings of school life: Action research for preservice teachers. *Curriculum and Teaching*, *27*(1), 67–79

Stewart, M. A. (2015). “My journey of hope and peace”: Learning from adolescent refugees’ lived experiences. *Journal of Adolescent & Adult Literacy*, *59*(2), 149-159

Stokar, H., Davis, L., Sinha, B., LaMarca, L., Harris, A., Hellum, K., & McCrea, K. T. (2017). “Love your love life”: Disadvantaged African American adolescents cocreate psychoeducational romantic and sexual health resources. *Social Work*, *62*(2), 146–155. [doi:10.1093/sw/swx012](https://academic.oup.com/sw/article-abstract/62/2/146/2993888?redirectedFrom=fulltext)

Straus, P. (2017). The Prime Time Games: It’s inclusion, but who’s including who? *Palaestra*, *31*(4), 32-37.

Subramaniam, M., Ahn, J., Waugh, A., Taylor, N. G., Druin, A., Fleischmann, K. R., & Walsh, G. (2013). The role of school librarians in enhancing science learning. *Journal of Librarianship and Information Science*, *47*(1), 3–16. [doi:10.1177/0961000613493920](https://journals.sagepub.com/doi/10.1177/0961000613493920)

Subramaniam, M., St Jean, B., Taylor, N. G., Kodama, C., Follman, R., & Casciotti, D. (2015). Bit by bit: Using design-based research to improve the health literacy of adolescents. *JMIR Research Protocols*, *4*(2). [doi:10.2196/resprot.4058](https://pubmed.ncbi.nlm.nih.gov/26025101/)

Symons, C., & Ponzio, C. (2019). Schools cannot do it alone: A community-based approach to refugee youth’s language development. *Journal of Research in Childhood Education*, *33*(1), 98–118. [doi:10.1080/02568543.2018.1531450](https://www.tandfonline.com/doi/full/10.1080/02568543.2018.1531450)

Thulin, E. J., Lee, D. B., Eisman, A. B., Reischl, T. M., Hutchison, P., Franzen, S., & Zimmerman, M. A. (2022). Longitudinal effects of Youth Empowerment Solutions: Preventing youth aggression and increasing prosocial behavior. *American Journal of Community Psychology*, *70*(1-2), 75-88. [doi:10.1002/ajcp.12577](https://onlinelibrary.wiley.com/doi/abs/10.1002/ajcp.12577)

Travis Jr., R., Gann, E., Crooke, A. H. D., & Jenkins, S. M. (2019). Hip hop, empowerment, and therapeutic beat-making: Potential solutions for summer learning loss, depression, and anxiety in youth. *Journal of Human Behavior in the Social Environment*, *29*(6), 744–765. [doi:10.1080/10911359.2019.1607646](https://www.tandfonline.com/doi/full/10.1080/10911359.2019.1607646)

Tucker-Raymond, E., Lewis, N., Moses, M., & Milner, C. (2016). Opting in and creating demand: Why young people choose to teach mathematics to each other. *Journal of Science Education and Technology*, *25*(6), 1025–1041. [doi:10.1007/s10956-016-9638-0](https://link.springer.com/article/10.1007/s10956-016-9638-0)

Ustach, E. (2020). Flexibility and fidelity in a drop-in, open-door art studio program. *Afterschool Matters*, *32*, 3-9.

Vera, E., Shriberg, D., Alves, A., Montes de Oca, J., Reker, K., Roche, M., Salgado, M., Stegmaier, J., Viellieu, V., Knoll, M., Adams, K., Diaz, Y., & Rau, E. (2016). Evaluating the impact of a summer dropout prevention program for incoming freshmen attending an under-resourced high school. *Preventing School Failure: Alternative Education for Children and Youth*, *60*(2), 161–171. [doi:10.1080/1045988x.2015.1063039](https://www.tandfonline.com/doi/full/10.1080/1045988X.2015.1063039)

Vickery, J. R. (2014). The role of after-school digital media clubs in closing participation gaps and expanding social networks. *Equity & Excellence in Education*, *47*(1), 78–95. [doi:10.1080/10665684.2013.866870](https://www.tandfonline.com/doi/full/10.1080/10665684.2013.866870)

Wallace Foundation, The. (2022a). *Youth perspectives on designing equitable out-of-school-time programs*. Wallace’s Considerations Series.

Wallace Foundation, The. (2022b). *From access to equity: Making out-of-school-time spaces meaningful for teens from marginalized communities*. Wallace’s Considerations Series.

Whalen, L., McCaughtry, N., Garn, A., Kulik, N., Centeio, E. E., Maljak, K., Kaseta, M., Shen, B., & Martin, J. (2016). Why inner-city high-school students attend after-school physical activity clubs. *Health Education Journal*, *75*(6), 639–651. [doi:10.1177/0017896915608885](https://journals.sagepub.com/doi/10.1177/0017896915608885)

Wrensford, G. E., Stewart, K.-A., & Hurley, M. M. (2019). A health professions pipeline for underrepresented students: Middle and high school initiatives. *Journal of Racial and Ethnic Health Disparities*, *6*(1), 207–213. [doi:10.1007/s40615-018-0515-9](https://pubmed.ncbi.nlm.nih.gov/30014447/)

Wroten, K., Reames, E. S., & Tuuri, G. (2012). Diabetes awareness of low-income middle school students participating in the Help a Friend, Help Yourself Youth Diabetes Awareness Education Program. *Journal of Extension*, *50*(1).

Wu, I.-C., Pease, R., & Maker, C. J. (2019). Students’ perceptions of a special program for developing exceptional talent in STEM. *Journal of Advanced Academics*, *30*(4), 474–499. [doi:10.1177/1932202X19864690](https://journals.sagepub.com/doi/full/10.1177/1932202X19864690)

Yazel-Smith, L., El-Mikati, H. K., Adjei, M., Haberlin-Pittz, K. M., Agnew, M., & Hannon, T. S. (2020). Integrating diabetes prevention education among teenagers involved in summer employment: Encouraging environments for health in adolescence (ENHANCE). *Journal of Community Health*. [doi:10.1007/s10900-020-00802-2](https://pubmed.ncbi.nlm.nih.gov/32146639/)

Zarrett, N., Skiles, B., Wilson, D. K., & McClintock, L. (2012). A qualitative study of staff’s perspectives on implementing an after school program promoting youth physical activity. *Evaluation and Program Planning*, *35*(3), 417–426. [doi:10.1016/j.evalprogplan.2011.12.003](https://pubmed.ncbi.nlm.nih.gov/22343428/)
